# The Power of Unrequited Love: The Parasocial Relationship, Trust, and Organizational Identification Between Middle-Level Managers and CEOs

**DOI:** 10.3389/fpsyg.2021.689511

**Published:** 2021-09-10

**Authors:** Youliang Liao, Bin Lin, Haiyan Zhou, Xi Yang

**Affiliations:** ^1^School of Business, Sun Yat-sen University, Guangzhou, China; ^2^Robert C. Vackar College of Business & Entrepreneurship, University of Texas Rio Grande Valley, Edinburg, TX, United States; ^3^NUS Business School, National University of Singapore, Singapore, Singapore; ^4^Center for Accounting, Finance and Institutions, Sun Yat-sen University, Guangzhou, China; ^5^China Institute for Small and Medium Enterprises, Zhejiang University of Technology, Hangzhou, China

**Keywords:** parasocial relationship, trust, CEO, middle-level manager, organizational identification

## Abstract

Previous studies have found that CEOs manage their firms through traditional methods such as leadership and management practices. In this study, we investigate how the parasocial relationship (PSR) between middle-level managers and CEOs affects the organizational trust and the organizational identification (OI) of middle managers. We find that the PSR between middle managers and CEOs has a positive effect on the OI of middle managers, which is mediated by the organizational trust of middle managers.

**Purpose:** Middle managers and CEOs are the key components of a firm and are crucial to firm strategies and control systems. Middle managers play a vital role in information transmission like in the organizational hierarchy while CEOs influence low-level employees through middle managers. In this study, we investigate how the PSR between middle managers and CEOs affects organizational trust and OI.

**Design/Methodology:** In this study, the data concerning OI, integrity perception, and organizational trust are derived from a survey conducted by the internal control research group of the China Securities Regulatory Commission (CSRC). The research group began the survey on September 5, 2014, for the firms listed in the A-share market, accounting firms with securities and future practice qualifications, and institutional investors through the accounting department of the CSRC, the Shanghai Stock Exchange, the Shenzhen Stock Exchange, and the Asset Management Association of China. The research group members surveyed 2,536 A-share firms listed on the Shanghai Stock Exchange and Shenzhen Stock Exchange. As of October 31, 2014, 2,154 sets of questionnaires with a total of 12,551 questionnaires were received, with a response rate of 84.95%. The financial and accounting data are from the China Stock Market and Accounting Research (CSMAR) database.

**Findings:** We find that the PSR between middle managers and CEOs has a positive effect on the OI of middle managers, which is mediated by the organizational trust of middle managers. This study extends the application of the parasocial interaction (PSI) theory, organizational trust theory, and social identity theory in listed firms.

**Practical Implication:** There are practical implications for internal relationship management, corporate governance, and performance management. CEOs should value the influence of organizational trust and improve his/her own social and work abilities on middle-level managers as the organizational trust of middle-level managers has a significant positive impact on their sense of responsibility, ethical behavior, organizational commitment, job satisfaction, and performance. CEOs should adopt various methods to influence different managers because organizational trust mediates the relationship between the PSR and OI.

**Originality/Value:** Our study is one of the first attempts to apply the PSI theory to the corporate world. Given the dynamics of present-day markets and changing stakeholder demands, there is little insight into how this relationship affects organizational health and functioning. Much less what a PSR between CEO and middle management looks like in practice. Our study attempts to fill the gap by investigating how CEOs might come to affect middle managers through their practices and behaviors.

## Introduction

A parasocial relationship (PSR) originates from the one-sided feelings of fans toward celebrities or superstars. Because PSR is a unilateral and a virtual relationship that emerges in the case of individuals not being able to obtain normal social interactions with a particular person, the type of relationship between CEOs and middle-level managers is also parasocial. Middle-level managers are the key components of a firm and are crucial to firm strategies and control systems, and, in particular, information transmission (Sminia and de Rond, 2012). Previous studies have found that CEOs manage their firms through traditional methods such as leadership and management practices (Yukl, 2008; Finkelstein et al., 2009; Schein and Schein, 2010; Ou et al., 2014). In this study, we investigate how the PSR between middle-level managers and CEOs affects the organizational trust of middle-level managers and the organizational identification (OI) of middle managers.

The influence of CEOs on employees is well-recognized by researchers and practitioners (Weitz and Bradford, 1999; Nath and Mahajan, 2011; Germann et al., 2015). However, with growing complexity in traditional organizational hierarchies, the hierarchical distance between CEOs and middle-level managers increases and the interaction between middle-level managers and CEOs drastically decreases, which mitigates the influence of CEOs on middle-level managers due to a lack of interaction and a weak relationship (Williams and Bargh, 2008). Consequently, how to deal with the weakening impact of CEOs resulting from a growing complexity in traditional organizational hierarchies is a fundamental challenge in corporate governance. Previous studies focusing on the relationship between employees and CEOs investigate the following: (1) the effect of characteristics of CEOs on employees such as motivation, communication style, power, and social influence of managers (Whitener et al., 1998; Rich, 2001); (2) the effect of characteristics of employees on performance such as incentives, personality, working style, and compensation (Cravens et al., 1993; Miao and Evans, 2014; Kissan and Alex, 2015); and (3) a moderating effect of environmental factors, such as organizational culture, competitive intensity, and market uncertainty, on the relationship between CEOs and employees (Ehrhart and Naumann, 2004; Fraenkel et al., 2016). These studies focus on the management strategies of CEOs but overlook the active feedback effect of employees (Agnihotri and Krush, 2015). With growing complexity in traditional organizational hierarchies, the effect of management strategy will gradually decay from the top to the bottom of the hierarchy. Therefore, it is critical to explore the initiatives of employees, including middle-level managers (Ehrhart and Naumann, 2004; Bush et al., 2017).

Middle managers are the key components of firm employees and play a pivotal role in any organization (Holmemo and Ingvaldsen, 2016; Lleo et al., 2017), who act as internal information intermediaries in firms (Katz and Kahn, 1978; Yang et al., 2010) and mediators between top management teams (TMT) and front-line employees (Wooldridge et al., 2008; Yang et al., 2010). They are important practitioners of firm strategies (Mantere, 2008), ensuring effective implementation of strategic plans and mitigating the obstacles of CEOs and front-line employees in the firm reform (Guo et al., 2017; Lampaki and Papadakis, 2018).

In normal management hierarchies, CEOs influence employees through other TMT members and middle managers with whom CEOs can impact (Ou et al., 2014). Previous studies report that CEOs influence middle-level managers and other subordinates with traditional approaches such as leadership, management practices, and organizational culture (Yukl, 2008; Finkelstein et al., 2009; Schein and Schein, 2010; Ou et al., 2014). Instead of focusing on the traditional approaches, we investigate the PSR between middle managers and CEOs given the importance of middle managers in this study. Given the dynamics of present-day markets and changing stakeholder demands, there is little insight into how this relationship affects organizational health and functioning. Much less what a PSR between CEO and middle management looks like in practice. Our study attempts to fill the gap by investigating how CEOs might come to affect middle managers through their practices and behaviors.

Parasocial interaction (PSI) theory provides a lens to explore the importance of fostering employee initiative (Rubin and Mchugh, 1987; Giles, 2002). PSI theory argues that in an interaction between the two parties, wishful thinking (i.e., perceived kindness) of one party toward another party can facilitate building a PSR (Rubin et al., 1985). Different from a traditional social relationship, a PSR is a one-sided virtual relationship, where one party initiates the relationship and another party is unaware of its existence (Rubin and Step, 2000). PSRs originate from the intimacy and identification for a receiver of initiator and are allowed to build a psychological connection (Perse and Rubin, 1989; Labrecque, 2014). A PSR prompts a receiver to conduct beneficial behavior toward the initiators (Perse and Rubin, 1989; Labrecque, 2014).

Extant literature on PSRs is based on new social media, such as Facebook, Twitter, Instagram, Snapchat, and Tumblr, which facilitate the formation of PSRs (e.g., Kim and Song, 2016). We argue that such PSRs could exist in the corporate world. When the growth of and an increase in the management hierarchy of a firm limits the interaction between CEOs and middle managers, the PSR is expected to become more crucial. For instance, star entrepreneurs such as Steven Jobs, Bill Gates, Warren Edward Buffett, Jack Ma, and Richard Liu can stimulate the workplace initiative of their employees. PSRs play an important role where middle and low-level managers can barely interact with these entrepreneurs but are still motivated by them. However, in the setting of internal relationships and corporate governance in the company, research on the effect of PSRs is still missing. Our study attempts to fill such a research gap. Based on the PSI theory, this study explores the effect of PSRs on OI. OI is the perceived degree of a stakeholder to which they are connected to and share the same values like an organization (Ashforth and Mael, 1989; Dutton et al., 1994; Pratt, 1998). We attempt to answer the following questions in this study: (1) How does the PSR between CEOs and middle managers affect the OI of middle managers? and (2) What is the mechanism through which the PSR affects OI?

In this study, we obtain the data concerning OI, integrity perception, and organizational trust from a survey conducted by the internal control research group of the China Securities Regulatory Commission (CSRC). The research team started to survey A-share listed companies through the China Securities Regulatory Commission (CSRC) on September 5, 2014, for the firms listed in the A-share market, accounting firms with securities and future practice qualifications, and institutional investors through the accounting department of the CSRC, the Shanghai Stock Exchange, the Shenzhen Stock Exchange, and the Asset Management Association of China. The research group members surveyed 2,536 A-share firms who are publicly listed on the Shanghai Stock Exchange and Shenzhen Stock Exchange. As of October 31, 2014, 2,154 sets of questionnaires with a total of 12,551 questionnaires were received, with a response rate of 84.95%. The questionnaire was filled in by senior and middle managers, such as CEO, chairman of the board, secretary of the board, financial department manager, auditing department manager, and the manager of the internal control department. The financial and accounting data are from the China Stock Market and Accounting Research (CSMAR) database. We find that the PSR between middle managers and CEOs is positively associated with the OI of middle managers. Further, we show that that relationship is mediated by organizational trust.

Our study makes theoretical contributions to the literature in the following ways. Firstly, our study is one of the first attempts to apply the PSI theory to the corporate world. Our study is different from the existing literature studies on PSRs, which is based on new social media, such as Facebook, Twitter, YouTube, Instagram, Snapchat, and Tumblr, to facilitate the formation of PSRs (e.g., Kim and Song, 2016). In previous studies, PSI theory was used to study the impact of the PSR or PSI of a multimedia platform on the attitude and behavior of consumers (e.g., Kim and Song, 2016; Yuksel and Labrecque, 2016; Gong and Li, 2017). In the relevant research on executives of the company, it is mainly the research on the executives of the company influencing the public through the mass media (e.g., Men and Tsai, 2016). None of these studies examined the PSR between the middle manager and CEO in a firm. Secondly, to add OI to the literature (e.g., Boivie et al., 2011; Lange et al., 2015), we reveal a mediating role of organizational trust between the PSR and the OI. Our study also contributes to the motivation literature (e.g., Rubin and Step, 2000) by documenting the role of the PSR in enhancing the organizational trust, OI, and initiatives of middle managers. For practitioners, our model sheds light on improving the PSR between middle managers and CEOs, encourages CEOs to motivate middle managers, and promotes the OI of middle managers. By strengthening the degree of OI in middle management positions, CEOs can motivate people in these roles and simultaneously improve the nature of PSRs in their organization, which has positive performance benefits.

## Theoretical Background

### PSR Between Middle Managers and CEOs

A PSR is characterized by an interest in persona and a persistent will to build emotional trust (Lim and Kim, 2011). For example, the PSR of an individual with a celebrity originates from appreciation and makes his or her trust evident by buying the products endorsed by the celebrity. Social interaction and communication between the two parties is a basic component of the life of an individual and a tool to form social relationships (Rubin and Mchugh, 1987). In traditional society, interpersonal interactions are bilateral; however, in a PSI, emotional dependence is a result of one-sided perception, which leads to a PSR (Rubin and Mchugh, 1987). A PSR originates from pseudo-intimacy in which personas express concern to manipulate others (Lim and Kim, 2011). For example, an audience is willing to build a PSR because he/she feels that the celebrity cares about him/his (Chen, 2014). Because celebrities are not aware of the relationship and do not provide feedback to an audience, this type of unilateral relationship is considered as a PSR (Hoffner, 1996). Based on the PSI theory, although an audience receives the same information (i.e., watching the same TV show, broadcast program, or speech from a conference) from the persona, different receivers may form PSRs with varying intensities, which in turn leads to varying cognition, attitudes, and behaviors (Ehrhart and Naumann, 2004).

According to the previous literature studies (i.e., Rubin et al., 1985; Dibble et al., 2016), we define the PSR between middle managers and CEOs as a persistent and an intimate relationship developed between middle managers and CEOs based on the one-sided perception of middle managers (Rubin et al., 1985). This PSR has four characteristics: (1) a one-sided relationship from middle managers to CEOs (Rubin et al., 1985) in which middle managers perceive the language and behavior of CEOs unilaterally and form an emotional bond to CEOs; (2) an illusionary experience (Hartmann and Goldhoorn, 2011) in which middle managers interpret the signals sent by CEOs and perceive the feeling of reciprocity with the consensus, attention, and adjustment of executives; (3) a long-term relationship (Dibble et al., 2016; Hoewe et al., 2020); and (4) a relationship similar to a real social relationship (Gleason et al., 2017; Tukachinsky and Stever, 2019), which is based on social attraction and can provide a feeling of friendship with CEOs (Perse and Rubin, 1989). According to the PSI theory, a PSR strengthens the obsession of information receivers with the information transmitters themselves. The PSR between middle managers and CEOs can affect their work enthusiasm (Tsai and Men, 2017). From the perspective of PSR formation, there are three categories of mechanisms underlying PSRs: information transmitters (CEOs), information receivers (middle managers), and the other factors influencing the perceptions of information receivers.

A PSR is based on the one-sided perception of middle managers on the information of the CEO. Like information transmitters, the antecedents of PSR of middle managers could be factors such as the self-disclosure (Kim and Song, 2016; Chung and Cho, 2017), social presence (Kim and Song, 2016), exposure (Horton and Wohl, 1956; Cohen, 2009; Bond, 2018), awareness, liveliness (Kim et al., 2016), competence, trustworthiness, goodwill, and care of CEOs (Tsiotsou, 2016). Previous studies have shown that the attractiveness of a TV host or actor strengthens the PSR of an audience with them (Conway and Rubin, 1991; Turner, 1993). The similarity between the conduct of information transmitters and information receivers is positively associated with their likeability (Duck and Barnes, 1992) and trust (Phua, 2016), thus enhancing PSR (Schiappa et al., 2007; Bond, 2018).

Individual heterogeneity affects the formation and strength of the PSR. The same behavior of a CEO can be interpreted differently by middle managers. Comparatively, information-sensitive individuals are more likely to form a PSR with CEOs when they express caring signals (Cravens et al., 1993). Self-esteem, self-efficacy, neuroticism, introversion, materialism, etc., of middle managers can affect their own PSR (Sun and Wu, 2012). When middle managers regard CEOs as “friends,” this intimacy perception may increase their job commitment (Rubin et al., 1985). Cohen (2004) expresses that demographic characteristics such as gender, age, and education affect the strength of a PSR (Cohen, 2004). Jin and Namkee (2009) show that game players with high interdependent self-construal are positively associated with the PSR of game players with their game avatars. Lim and Kim (2011) find that the feeling of loneliness of customers positively predicts the PSR between them and TV shopping hosts.

Other factors influencing the perceptions of information receivers can also affect PSRs. For instance, Rubin and Mchugh (1987) document that how audiences perceive the strength of PSR corresponding with the information credibility of radio hosts. Biel and Bridgwater (1990) indicate that when the perceived relevancy of TV audiences between their own needs and commercial products is strong, there is more participation from the audiences and a stronger PSR is present. An intimate PSR will emerge when middle managers perceive the care from CEOs (Tsai and Men, 2017). Rubin and Step (2000) and Ehrhart and Naumann (2004) show that the PSR can alter the cognition, attitude, and behavior of the information receiver by increasing the perception of information reliability.

The social information processing theory argues that the attitude and behavior of an individual are affected by the information received from others (Salancik and Pfeffer, 1978; Ou et al., 2014). PSRs affect the cognition, attitude, and behavior of information receivers (Ehrhart and Naumann, 2004). Rubin and Mchugh (1987) empirically examine the PSR between radio hosts and their audiences, finding that the PSR is positively related to the radio exposure of an audience on the radio station, information acquisition from radio hosts, and positive attitudes and behaviors. Ballantine and Martin (2005) argue that with a stronger PSR between media personas and media users is, the users are more likely to buy the products promoted by the media personas in online communities. Those findings explain why companies pay a large sum of money to celebrities as endorsement fees (Song and George, 2008). Labrecque (2014) finds that the PSR between information receivers in online brand communities is positively associated with the willingness to share information and brand loyalty. Thorson and Rodgers (2006) analyze the interaction between college students and political candidates on blogs and find that the PSR between college students (the information receivers) and political candidates (the information transmitters) positively affects the positive attitude of information receivers toward candidates, and the willingness to vote.

### Organizational Identification

Organizational identification is an integral part of a firm, which makes it prominently different from the market along with other factors such as cooperation, communication, learning, and loyalty (Kogut and Zander, 1996). The social identity theory provides a theoretical foundation for OI (Elsbach, 1979). Social identity originates from social norms, social situations, and social categories (Akerlof and Kranton, 2005). Firstly, social norms govern how people should behave (Pareto, 1980). Secondly, social situations influence how people internalize norms and then guide their behavior accordingly, as well as the situation itself—that is, when, where, how, and between whom a transaction takes place. Finally, social categories are used to describe the types of people (e.g., gender or ethnicity) and are critical to behaviors as people often consciously think of themselves in terms of social categories to a greater or lesser extent (Akerlof and Kranton, 2005). Previous studies argue that social categories are crucial for the behavior of people because they classify themselves into that given social category (Akerlof and Kranton, 2005). While these clearly articulate the consequences of PSR, none of the studies, however, attempt to illustrate linkages between potential or expected implications for middle management.

Identity is a self-image of a person of who he/she is, based on his/her social categories (Akerlof and Kranton, 2005). Accordingly, OI is the perceived degree of a stakeholder to which they are connected to and share the same values as the organization (Ashforth and Mael, 1989; Dutton et al., 1994; Pratt, 1998; Ashforth et al., 2008). OI is also described as a process of self-categorization (Dutton et al., 1994). The connotation is that the perception and feeling of belonging to an organization of an individual or of sharing the fate of an organization is a process in which a person uses his/her identity as an organization member to define his/herself (Mael and Ashforth, 1992). Therefore, we define the OI of middle managers as the cognition, emotional connection, and feeling of belonging to the organization of middle managers, and the perceived status as an organizational member (Mael and Ashforth, 1992).

The relationship between individuals and the other members of an organization will affect OI (Sluss and Ashforth, 2008). In the corporate setting, the PSRs between CEOs and middle-level managers could affect the OI of middle managers. As identification is an effective approach to motivate and can substitute for lucrative or material incentives (Akerlof and Kranton, 2005), such a link would benefit a firm when an identification is developed and people integrate their belief in firms into their own identities. The PSRs between CEOs and middle managers can enhance the identification process, when PSRs make the middle managers to believe that the group of an individual is more unique and favorable than other groups and his organizational identity is stronger (Ashforth and Mael, 1989). In addition, the long-term PSRs between CEOs and middle managers can enhance the identification process, as a salient, central, and long-lasting group, which also enhances the identity of its members (Albert and Whetten, 1985; Ashforth and Mael, 1989; Dutton et al., 1994).

### Organizational Trust

Trust is the willingness of an individual to accept positive expectations based on the intentions and actions of others (Mayer et al., 1995). Positive expectations are based on the perception and evaluation of the extent to which individuals trust others, based on the emotional reaction made to others by individuals (Williams and Anderson, 1991; McAllister, 1995). The contents of perception and evaluation are classified into two categories: capability and goodwill. Capability indicates the skills necessary to fulfill a task, whereas goodwill indicates the willingness of the trusted object to fulfill a task (Agnihotri and Krush, 2015). Trust implies the willingness to bear risks resulting from the possible opportunistic behavior of a trusted object. In organizational management, trust is an intangible asset, which alleviates the transaction costs, facilitates the implementation of various organizational plans, strategies, and activities, and improves the spontaneous communication and cooperation of members in an organization (Agnihotri and Krush, 2015).

**Figure 1:**
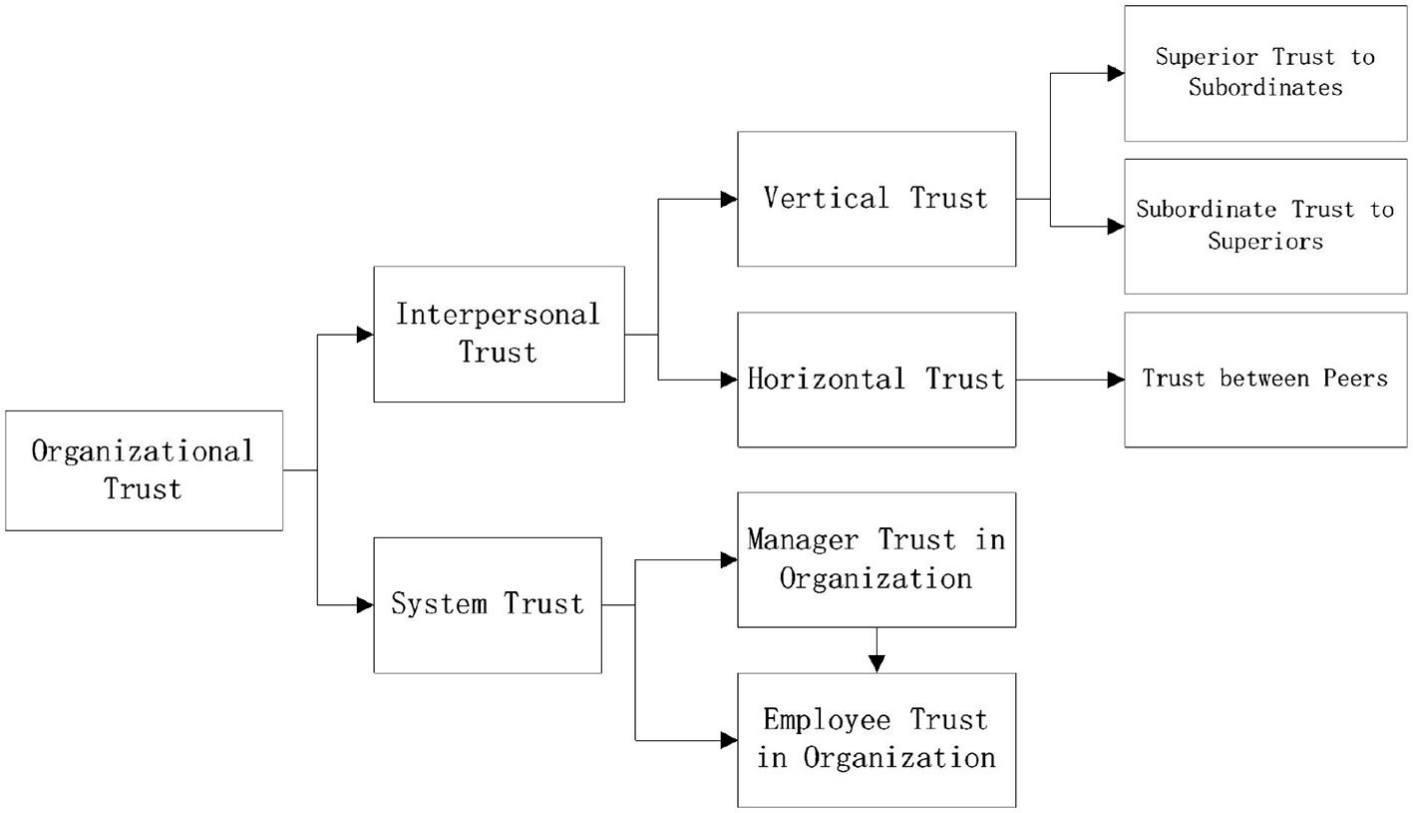
Organizational trust definition.

Luhmann (2005) categorizes trust, in terms of trusted objects, into “individual trust” and “systematic trust.” Individual trust defines the trusted objects as individuals, whereas systematic trust defines them as organizations. Organizational trust includes the trust between peers and the mutual trust between subordinates and superiors, or organizations (Costigan et al., 1998). In summary, we classify organizational trust as shown in Figure 1.

Integrating the characteristics of individual trust and organizational trust, we define the organizational trust of middle managers as the willingness of middle managers to accept positive expectations based on the intentions and actions of CEOs (Mayer et al., 1995). Consequently, we use the perception of middle managers on an improvement of the trust in an organizational environment to proxy for the organizational trust of middle managers.

The organizational trust of employees predicts their responsibility, ethical behavior, organizational commitment, job satisfaction, and performance (Williams and Anderson, 1991; Agnihotri and Krush, 2015). In addition, organizations possess personified characteristics, and CEOs are considered as the representatives of organizational personification (Hambrick and Mason, 1984). Moreover, the higher the status of the trusted object is, the more likely employees will attribute to the behavior of the trusted object to organizational objectives (Eisenberger et al., 2004). Because executives are typical representatives of organizational personification and organizational intent (Hambrick and Mason, 1984; Eisenberger et al., 2004), employee trust in executives can be considered as employee trust in the organization. The magnitude of the trust of employees in the CEOs is affected by the quality of the relationship between them—the higher the intimacy level, the stronger the trust (Lin, 2010; Chen, 2014). A PSR, a long-lasting, one-sided intimacy relationship will make middle managers to regard CEOs as friends and enhance the one-sided intimacy and commitment in the relationship (Rubin and Mchugh, 1987; Rubin and Step, 2000). Therefore, the PSR of middle managers can be an antecedent of trust and improve their organizational trust (Figure 1) (Allison et al., 2016).

## Hypothesis Development

### The Influence Mechanism of PSR

Based on the PSI theory, the PSR emerges as the two parties involved rarely interact or communicate directly; instead, one party develops an emotional bond voluntarily (Rubin and Mchugh, 1987). This relationship is asymmetrical (Rubin et al., 1985). The PSR that is underscored with affection indicates a closer psychological distance between the two parties involved, and that closer distance can affect cognition and behavior.

When faced with many middle managers, the CEO cannot maintain direct communication with each one of them. However, middle managers have normal social, emotional, and professional needs from CEOs. Additionally, the perception of CEOs as “friends” is beneficial for work initiatives (Weitz and Bradford, 1999).

When middle managers cannot easily build a friendship with CEOs, a PSR may be an effective alternative (Perse and Rubin, 1989). CEOs convey the information on subordinate care, work plans, etc., to middle managers so that they may interpret that information and generate emotional resonance (Rubin and Mchugh, 1987). This will influence a unilateral perception of middle managers on the friendship of CEOs. The PSR requires the unilateral approval of middle managers and exerts a positive influence on them (Lim and Kim, 2011).

The PSR between middle managers and CEOs has three stages. Stage one indicates the affection of middle managers for CEOs, which is the foundation of a PSR. Stage two indicates the identification of middle managers for CEOs, by which the skills and visions of CEOs induce resonance (Rubin and Step, 2000). Stage three implies that middle managers regard CEOs as not only leaders but also friends (Lim and Kim, 2011). The three stages of emotional connection are all premises of OI (Schaubroeck et al., 2013).

Organizational identification is the degree to which individuals self-define in relation to the organization (Ashforth et al., 2008). The relationship between the colleagues in an organization will affect OI (Sluss and Ashforth, 2008). A PSR shares similar characteristics with a normal social relationship, such as motivation, communication style, power, and social influence (Whitener et al., 1998; Rich, 2001), so it is highly likely to affect the OI of an individual. The good relationship between superiors and subordinates helps to improve the OI of subordinates (Morgan et al., 2004; Katrinli et al., 2008). Horizontal partnerships could influence the OI of an employee with his/her employer (Cornwell et al., 2018). Effective organizational communication atmosphere can significantly promote the OI of employees (Smidts et al., 2001). Additionally, a PSR leads to the emotional connection of an individual, the positive effect of which can improve OI (Schaubroeck et al., 2013). Hence, PSRs can have a positive association with OI. Thus, we propose the following hypothesis:

*H1: The PSR of middle managers to CEOs is positively related with middle managers' OI*.

In addition to increasing the commitment of the information receiver (Rubin et al., 1985; Grant et al., 1991), PSRs also generate organizational trust in the objects (Fritchie and Johnson, 2003; Labrecque, 2014). Tsiotsou (2016) finds that the PSR of an individual in a consumer community affects brand trust. Individuals who trust in the brand or organization exhibit a positive attitude, including satisfaction, organizational commitment, and loyalty (Ballester and Alemán, 2001; Tsiotsou, 2016). Chung and Cho (2017) show that the PSR between consumers and celebrities positively predicts the perception of consumers on trust in celebrities. Previous studies indicate the PSR of an individual as being positively related to trust. Furthermore, the trust of an employee in coworkers and managers is positively associated with OI (Schaubroeck et al., 2013).

**Figure 2:**
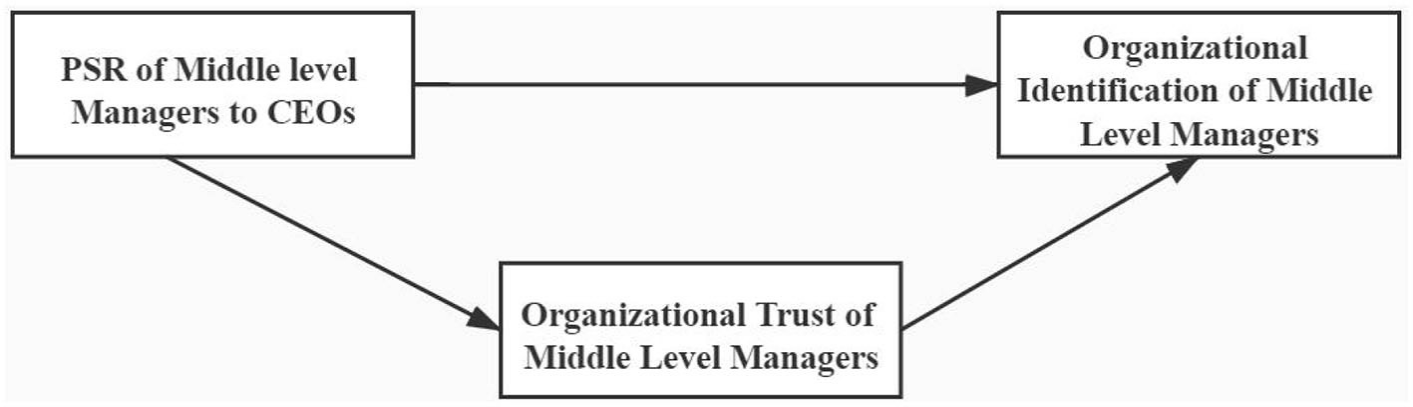
Conceptual model.

Consequently, we have the following hypotheses (Figure 2):

*H2: The PSR of middle managers to CEOs is positively related with middle managers' organizational trust*.
*H3: Middle managers' organizational trust mediates the PSR of middle managers to CEOs and the middle managers' OI*.

## Research Design

### Data and Sample Selection

In this study, the data concerning OI, environment integrity perception, and organizational trust are derived from a survey conducted by the internal control research group of the CSRC. The questionnaire was designed by Chinese and international researchers based on relevant references. The research group began a survey on September 5, 2014, for the firms listed in A-share market, accounting firms with securities and future practice qualifications, and institutional investors through the accounting department of the CSRC, the Shanghai Stock Exchange, the Shenzhen Stock Exchange, and the Asset Management Association of China. The research group members surveyed 2,536 A-share listed firms. As of October 31, 2014, 2,154 sets of questionnaires with a total of 12,551 questionnaires were received, with a response rate of 84.95%. The financial and accounting data are all from the CSMAR database.

According to previous studies, we process the data by: (1) excluding the samples with severely missing data in the questionnaire, (2) supplementing any remaining missing values in the questionnaire by the serial mean substitution method, (3) deleting samples of listed companies in the financial industry, and (4) dropping the missing values of the combined data of questionnaire and CSMAR. Because the data of OI, integrity perception, and organizational trust are from self-reported surveys, only the data from 2014 are available. Finally, we obtain 1,568 firm observations.

### Key Variables and Measures

#### Dependent Variable

*Organizational Identification*: we use the six-item scale developed by Mael and Ashforth (1992). For example, “I think the success of my company is the success of mine.” Each question is measured by a five-point Likert scale. A higher score indicates a higher level of OI.

#### Independent Variables

*Parasocial Relationship*: following the models of Miles and Snow (2003) and Ittner et al. (1997) and the definition of the PSR (Hartmann and Goldhoorn, 2011), we use the sum of the two absolute values of the difference between the environment integrity perceptions of middle managers and CEOs and the difference between the OI of middle managers and CEOs to act as a proxy for the PSR between middle managers and CEOs as shown in Equation (1). According to the definition of environmental integrity perception and organizational identification (Ashforth and Mael, 1989), the difference in environmental integrity perception and organizational identification between CEO and middle-level managers can reflect the sense of reciprocity in consensus, attention, and adjustment between them outside and inside the organization. A greater sum of the two absolute values indicates a greater discrepancy of environment integrity perceptions and organizational identities between the middle managers and the CEOs. That is, a greater discrepancy between the shared feeling of reciprocity of middle managers and CEOs with the consensus, attention, and adjustment indicates a weaker PSR. The calculation equation is as follows:


(1) $$\begin{array}{l} PSR=|INTEGRITY_{m}-INTEGRITY_{c}|+|IDEN_{m}-IDEN_{c}| \end{array}$$

where *PSR* represents the parasocial relationship between middle managers and CEOs, *INTEGRITY*_*m*_ represents the environment integrity perception of middle managers, *INTEGRITY*_*c*_ represents the environment integrity perception of CEOs, *IDEN*_*m*_ represents the OI of middle managers, and *IDEN*_*c*_ represents the OI of CEOs.

*Integrity Perception*: the environmental integrity perception scale is designed by an expert group with reference to the previous classic literature (Knack and Keefer, 1997; Porta et al., 1997). Environmental integrity perception directly measures the perception of the integrity of CEOs and department managers regarding industry and region using a five-point Likert scale. A higher score indicates a higher perception of outside environmental integrity.


(2) $$\begin{array}{l} PSR\_IC=|IDEN\_IC-IDEN\_CEO|\\ +|IC\_INTEGRITY-CEO\_INTEGRITY| \end{array}$$

(3) $$\begin{array}{l} PSR\_FINANCE=|IDEN\_FINANCE-IDEN\_CEO|\\ +|FINANCE\_INTEGRITY-CEO\_INTEGRITY| \end{array}$$

(4) $$\begin{array}{l} PSR\_AUDIT=|IDEN\_AUDIT-IDEN\_CEO|\\ +|AUDIT\_INTEGRITY-CEO\_INTEGRITY| \end{array}$$

where *PSR_IC, PSR_FINANCE*, and *PSR_AUDIT* represent the PSR between internal control manager and CEOs, financial managers and CEOs, audit managers and CEOs, respectively. *IDEN_CEO, IDEN_IC, IDEN_FINANCE*, and *IDEN_AUDIT* represent the OI of CEOs, internal control managers, financial managers, and internal auditing managers, respectively. *CEO_INTEGRITY, IC_INTEGRITY, FINANCE_INTEGRITY*, and *AUDIT_INTEGRITY* represent the integrity perception of CEOs, internal control managers, financial managers, and audit managers, respectively.

#### Mediator

*Organizational Trust of Middle Managers*: We use the evaluation of department managers on the improvement of organizational credibility as a proxy for the organizational trust of middle managers, measured by a three-point scale. The question is: “Compared with the previous year, has the extent of which stakeholders' integrity improved?” A high score implies a high level of organizational trust. *TRUST_IC, TRUST_FINANCE*, and *TRUST_AUDIT* represent the organizational trust of internal control managers, financial managers, and audit managers, respectively.

#### Control Variables

The data for the control variables are collected from the CSMAR database. Control variables include *SOE, GROWTH, INVENTORY, SIZE, LOSS, AUDITOR_RESIGN, AUDITOR, DUAL, FIRST, MAO, INDEPEN, MANSHARE, TRADE, ROA*, and Σ*INDUSTRY*. Detailed definitions of all variables are shown in Table 1.

**Table 1:** Definitions of variables.

**Symbol**	**Variables**	**Definitions**
PSR	Parasocial relationship	We use the absolute value of the sum of the difference between the middle managers' and CEOs' perceptions of integrity on environment and the difference between the middle managers' and CEOs' organizational identification to proxy for the PSR between middle managers and CEOs.
TRUST	Organizational trust	We use department managers' evaluation of the improvement of organizational credibility as the proxy for organizational trust of middle managers, measured by a three-point scale. we use the six-item scale developed by Mael and Ashforth (1992).
IDEN	Organizational identification	*IDEN_CEO. IDEN_IC, IDEN_FINANCE*, and *IDEN_AUDIT* represent the organizational identification of CEOs, internal control managers, financial managers, and internal auditing managers, respectively.
SOE	State-owned firm	Indicator variable equal to 1 for state-owned firms and 0 otherwise.
GROWTH	Firm growth	Revenue growth rate.
INVESTORY	Inventory to total assets ratio	Inventory divided by total assets.
SIZE	Business scale	Natural logarithm of total assets.
LOSS	Loss	Indicator variable equal to 1 if net profit is negative and 0 otherwise.
AUDITOR_RESIGN	Auditor change	Indicator variable equal to 1 if external auditor is different from the one in previous year.
AUDITOR	Big four	External auditor is one of the Big Four CPA firms.
DUAL	Two positions	Dummy variable which is equal to 1 if Chairman and CEO are the same person and 0 otherwise.
FIRST	The shareholding ratio of the largest shareholder	The largest shareholder's holdings in percentage.
MAO	Audit opinions	Indicator variable equal to 1 for unqualified opinion and 0 otherwise.
INDEPEN	Ratio of independent directors	The ratio of independent board members.
MANSHARE	Management shareholding ratio	The percentage of shares held by executives.
TRADE	Stock liquidity	Average monthly trading volume divided by number of shares outstanding.
ROA	Return on assets	Net profit divided by average balance of total assets.
INDUSTRY	Industry	The industry classification is based on the 2012 industry classification of the China Securities Regulatory Commission.

#### Empirical Analysis

The regression model is as follows:


(5) $$\begin{array}{l} IDEN_{i,t}=\beta_{0}+\beta_{1}PSR_{i,t}+\beta_{2}TRUST_{i,t}+\beta_{3}GROWTH_{i,t}\\ +\beta_{4}SOE_{i,t}+\beta_{5}SIZE_{i,t}+\beta_{6}LOSS_{i,t}+\beta_{7}AUDITOR\_RESIGN_{i,t}\\ +\beta_{8}AUDITOR_{i,t}+\beta_{9}DUAL_{i,t}+\beta_{10}FIRST_{i,t}+\beta_{11}MAO_{i,t}\\ +\beta_{12}INDEPEN_{i,t}+\beta_{13}MANSHARE_{i,t}+\beta_{14}TRADE_{i,t}\\ +\beta_{17}INVESTORY_{i,t}+\beta_{16}ROA_{i,t}+\varepsilon_{i} \end{array}$$

In Equation (5), *IDEN* represents the OI, *PSR* represents the parasocial relationship, and *TRUST* represents the organizational trust. In the regression analysis, we include the control variables given in Table 1.

## Empirical Results and Analysis

### Descriptive Statistics

Table 2 provides the descriptive statistics for all variables. OI and trust are measured by a five- and three-point scale, respectively, so the maximum and minimum values of OI (organizational trust) are 5 and 1 (3 and 1). However, because some missing values are interpolated with the series MEAN, the minimum values and/or medians for some variables are not integers. Consequently, the mean (median) OI of CEO and department managers range from 4.20 to 4.27 (4–4.33). The average OI of all middle-level supervisors is high. Additionally, the SD is moderate and ranges from 0.55 to 0.64. The PSR of CEOs and department managers is between 0.85 and 0.95, on average. The SD is high at about 0.75. This could indicate the nature to which PSRs are experienced is highly variable and dependent on both personal and contextual factors. The mean of organizational trust ranges between 2.44 and 2.46 with a median of 2, indicating that CEOs and department managers have high organizational trust. The SD is also high at about 0.5.

**Table 2:** Descriptive statistics.

**Var Name**	**Obs**	**Mean**	**SD**	**Min**	**Median**	**Max**
IDEN_CEO	1,505	4.26	0.64	1	4.33	5
IDEN_IC	589	4.20	0.55	2.33	4	5
IDEN_FINANCE	1,494	4.27	0.55	2	4.17	5
IDEN_AUDIT	1,214	4.20	0.61	1	4	5
PSR_IC	424	0.95	0.75	0	0.92	4
PSR_FINANCE	1,105	0.85	0.74	0	0.83	4.33
PSR_AUDIT	902	0.93	0.75	0	1	5
TRUST_IC	512	2.45	0.51	1	2	3
TRUST_FINANCE	1,272	2.44	0.52	1	2	3
TRUST_AUDIT	1,041	2.46	0.50	1	2	3
GROWTH	1,568	0.17	0.61	−0.91	0.09	12.46
INVENTORY	1,568	0.14	0.11	0	0.12	0.78
SIZE	1,568	21.95	1.18	17.88	21.77	27.55
LOSS	1,568	0.09	0.29	0	0	1
AUDITOR_RESIGN	1,568	0.06	0.24	0	0	1
AUDITOR	1,568	0.04	0.20	0	0	1
SOE	1,568	0.31	0.46	0	0	1
DUAL	1,568	0.29	0.45	0	0	1
FIRST	1,568	34.71	14.59	3.62	33.11	85.04
MAO	1,568	0.02	0.16	0	0	1
INDEPEN	1,568	0.37	0.05	0.23	0.33	0.67
MANSHARE	1,568	0.16	0.21	0	0.019	0.81
TRADE	1,568	7.81	11.21	0.02	4.68	105.34
ROA	1,568	0.04	0.07	−0.78	0.04	0.96

Table 3 displays the univariate analysis results of state-owned and non-state-owned firm samples. In each sample, we show the mean comparison results of variables for each department. Consistent with our prediction, the PSR and OI of middle managers are not significantly different in state-owned and non-state-owned firms. However, the organizational trust in state-owned firms is lower than that of non-state-owned firms. State-owned firms are more likely to have a loss and perform worse than their non-state-owned counterparts. They are also more likely to hire a Big Four auditor firm and are less likely to change auditor firms. In addition, they have a larger size, higher market valuations, lower stock liquidities, less unqualified opinions, higher concentration on the largest shareholder, less shares held by the management, lower proportions of independent directors, and a higher inventory ratio percentage. Firm characteristics are consistent with the literature.

**Table 3:** The *t*-test between state-owed and non-state-owned firms.

**Department**	**Internal control**			**Finance**			**Audit**		
**Ownership and sample size**	**Non-state** **(161)**	**State-owned** **(263)**	***T*-test**	**Non-state** **(770)**	**State-owned** **(335)**	***T*-test**	**Non-state** **(693)**	**State-owned** **(209)**	***T*-test**
**Var name**	**Mean**	**Mean**	**Mean-diff**	**Mean**	**Mean**		**Mean**	**Mean**	**Mean-diff**
IDEN	4.231	4.181	0.050	4.292	4.262	0.030	4.233	4.242	−0.009
PSR	0.910	0.969	−0.059	0.838	0.887	−0.048	0.921	0.953	−0.032
TRUST	2.512	2.422	0.090^*^	2.464	2.431	0.032	2.495	2.423	0.072^*^
GROWTH	0.236	0.049	0.187^**^	0.204	0.062	0.142^***^	0.192	0.078	0.114^***^
INVENTORY	0.149	0.158	−0.009	0.136	0.161	−0.025^***^	0.134	0.165	−0.032^***^
SIZE	22.330	22.807	−0.477^***^	21.630	22.622	−0.993^***^	21.603	22.642	−1.039^***^
LOSS	0.068	0.144	−0.076^**^	0.065	0.134	−0.069^***^	0.051	0.163	−0.112^***^
AUDITOR_R~N	0.043	0.087	−0.044^*^	0.040	0.099	−0.058^***^	0.033	0.086	−0.053^***^
AUDITOR	0.056	0.095	−0.039	0.018	0.081	−0.062^***^	0.020	0.096	−0.075^***^
DUAL	0.273	0.087	0.186^***^	0.369	0.099	0.270^***^	0.351	0.105	0.245^***^
FIRST	30.964	39.732	−8.768^***^	33.082	38.791	−5.709^***^	32.967	38.244	−5.277^***^
MAO	0.019	0.034	−0.016	0.021	0.027	−0.006	0.017	0.029	−0.011
INDEPEN	0.373	0.367	0.006	0.377	0.366	0.011^***^	0.376	0.367	0.009^**^
MANSHARE	0.067	0.001	0.066^***^	0.230	0.008	0.222^***^	0.253	0.009	0.244^***^
TRADE	4.873	4.055	0.818^*^	9.616	4.495	5.121^***^	9.671	4.848	4.823^***^
ROA	0.044	0.024	0.020^***^	0.048	0.029	0.020^***^	0.051	0.024	0.027^***^

*, **, and *** *indicates a significance level at 10, 5, and 1%, respectively*.

### Reliability and Validity

The reliability of the measurement scale and questionnaire is evaluated with Cronbach's α. A large Cronbach's α value indicates that the scale is highly reliable. The validity of the scale includes construct validity and convergent validity. This study applies a confirmatory factor analysis (CFA) to examine the construct validity of the measurement scale. Standardized factor loading, composite reliability, and average variance extracted (AVE) are used to examine the construct validity and convergent validity of the scale. Table 1 shows the definitions of variables. The total reliability (Cronbach's α value) of the variables from the questionnaire is 0.937. The Cronbach α of the OI (integrity perception) [organizational trust] of CEOs and department managers is between 0.95 and 0.99 (0.91 and 0.99) [0.97 and 0.99]. The Cronbach α of all is >0.7, even reaching 0.9 (Nunally, 1978). Thus, both scale and questionnaire are quite reliable.

The results of CFA show that the factor loading of each variable is >0.5 and the contrast validity (CR) of OI, integrity, and organizational trust of CEO and department managers is between 0.90 and 0.99, 0.69 and 0.79, and 0.76 and 0.79, respectively, which meets the requirement of 0.6 (Bagozzi and Yi, 1988). The AVE of OI, integrity, and organizational trust of CEOs and department managers is between 0.61 and 0.65, 0.52 and 0.65, and 0.61 and 0.65, respectively, which meets the requirement of 0.5 (Bagozzi and Yi, 1988). A large CR or AVE value indicates that the measurement scale possesses high validity. The results of CFA indicate that the construct validity, composite validity, and convergent validity of the scale and questionnaire in this study are high. The results are shown in Table 4.

**Table 4:** Reliability and validity test of scale.

**Variable**	**Cronbach's** ***α***	
Organizational identification (middles and CEOs)	0.95–0.99	
Environment integrity perception (middles and CEOs)	0.91–0.99	
Organizational trust (middles and CEOs)	0.97–0.99	
**Variable**	**CR**	**AVE**
Organizational identification (middles and CEOs)	0.90–0.99	0.61–0.65
Environment integrity perception (middles and CEOs)	0.69–0.79	0.52–0.65
Organizational trust (middles and CEOs)	0.76–0.79	0.61–0.65

### Correlation Analysis

The correlation analysis in Tables 5–7 shows that *PSR* is significantly negatively correlated with *IDEN* and is significantly negatively correlated with *TRUST*. According to the measure of *PSR*, a larger *PSR* indicates a weaker PSR. This indicates that OI is significantly positively correlated with *PSR* and also with organizational trust. The results are consistent with H1 and H2.

**Table 5:** Correlation analysis: internal control managers.

	**(1)**	**(2)**	**(3)**	**(4)**	**(5)**	**(6)**	**(7)**	**(8)**	**(9)**	**(10)**	**(11)**	**(12)**	**(13)**	**(14)**	**(15)**	**(16)**	**(17)**
IDEN_IC	1																
TRUST_IC	0.15^*^	1															
PSR_IC	−0.10^*^	−0.19^*^	1														
GROWTH	0.04	0.06	−0.06	1													
INVENTORY	−0.05	−0.01	−0.01	−0.03	1												
SIZE	−0.01	0.07	−0.17^*^	0.00	0.12^*^	1											
LOSS	−0.00	−0.03	−0.07	−0.13^*^	0.01	−0.02	1										
AUDITOR_RESIGN	0.05	−0.00	0.07	0.15^*^	−0.01	0.06^*^	0.02	1									
AUDITOR	−0.03	0.01	0.01	−0.03	−0.01	0.39^*^	−0.02	0.07^*^	1								
SOE	−0.04	−0.09^*^	0.04	−0.11^*^	0.11^*^	0.39^*^	0.13^*^	0.09^*^	0.15^*^	1							
DUAL	0.03	0.03	0.03	0.01	−0.07^*^	−0.16^*^	−0.04	−0.03	−0.07^*^	−0.28^*^	1						
FIRST	0.01	−0.02	−0.06	−0.03	0.05	0.22^*^	−0.03	0.07^*^	0.14^*^	0.19^*^	−0.04	1					
MAO	0.04	0.02	−0.07	−0.06^*^	−0.01	−0.01	0.19^*^	0.03	−0.01	0.05^*^	−0.00	−0.02	1				
INDEPEN	−0.00	0.08	−0.01	−0.00	0.01	−0.05	0.01	0.01	0.03	−0.09^*^	0.12^*^	0.07^*^	0.03	1			
MANSHARE	0.05	0.05	0.02	0.08^*^	−0.11^*^	−0.36^*^	−0.12^*^	−0.03	−0.12^*^	−0.48^*^	0.23^*^	−0.10^*^	−0.06^*^	0.10^*^	1		
TRADE	0.04	−0.03	0.09	0.05	−0.09^*^	−0.33^*^	−0.08^*^	0.02	−0.08^*^	−0.20^*^	0.11^*^	−0.04	−0.05	0.05	0.34^*^	1	
ROA	0.03	0.09	0.07	0.26^*^	−0.10^*^	0.02	−0.52^*^	0.04	0.04	−0.16^*^	0.05^*^	0.03	−0.30^*^	−0.03	0.17^*^	−0.14^*^	1

*Lower-triangular cells report Pearson's correlation coefficients, upper-triangular cells are Spearman's rank correlation*.

* *p < 0.05*.

*IDEN, Organizational identification; PSR, Parasocial relationship; TRUST, Integrity perception*.

*The larger the PSR, the weaker the PSR between the department heads and the CEOs*.

**Table 6:** Correlation analysis: financial managers.

	**(1)**	**(2)**	**(3)**	**(4)**	**(5)**	**(6)**	**(7)**	**(8)**	**(9)**	**(10)**	**(11)**	**(12)**	**(13)**	**(14)**	**(15)**	**(16)**	**(17)**
IDEN_FINANCE	1																
TRUST_FINANCE	0.10^*^	1															
PSR_FINANCE	−0.10^*^	−0.15^*^	1														
GROWTH	0.00	0.06^*^	−0.05	1													
INVENTORY	−0.02	−0.01	0.02	−0.03	1												
SIZE	−0.01	0.02	−0.06^*^	0.00	0.12^*^	1											
LOSS	−0.01	−0.07^*^	0.02	−0.13^*^	0.01	−0.02	1										
AUDITOR_RESIGN	0.01	−0.02	−0.05	0.15^*^	−0.01	0.06^*^	0.02	1									
AUDITOR	−0.02	0.04	−0.02	−0.03	−0.01	0.39^*^	−0.02	0.07^*^	1								
SOE	0.01	−0.04	0.03	−0.11^*^	0.11^*^	0.39^*^	0.13^*^	0.09^*^	0.15^*^	1							
DUAL	0.05	−0.01	0.00	0.01	−0.07^*^	−0.16^*^	−0.04	−0.03	−0.07^*^	−0.28^*^	1						
FIRST	0.01	0.02	−0.02	−0.03	0.05	0.22^*^	−0.03	0.07^*^	0.14^*^	0.19^*^	−0.04	1					
MAO	0.04	0.02	−0.05	−0.06^*^	−0.01	−0.01	0.19^*^	0.03	−0.01	0.05^*^	−0.00	−0.02	1				
INDEPEN	0.03	−0.01	0.02	−0.00	0.01	−0.05	0.01	0.01	0.03	−0.09^*^	0.12^*^	0.07^*^	0.03	1			
MANSHARE	−0.01	0.02	−0.04	0.08^*^	−0.11^*^	−0.36^*^	−0.12^*^	−0.03	−0.12^*^	−0.48^*^	0.23^*^	−0.10^*^	−0.06^*^	0.10^*^	1		
TRADE	0.02	0.01	−0.01	0.05	−0.09^*^	−0.33^*^	−0.08^*^	0.02	−0.08^*^	−0.20^*^	0.11^*^	−0.04	−0.05	0.03	0.34^*^	1	
ROA	−0.00	0.09^*^	−0.02	0.26^*^	−0.10^*^	0.02	−0.52^*^	0.04	0.04	−0.16^*^	0.05^*^	0.03	−0.30^*^	−0.03	0.17^*^	−0.14^*^	1

*Lower-triangular cells report Pearson's correlation coefficients, upper-triangular cells are Spearman's rank correlation*.

* *p < 0.05*.

*IDEN, Organizational identification; PSR, Parasocial relationship; TRUST, Integrity perception*.

*The larger the PSR, the weaker the PSR between the department heads and the CEOs*.

**Table 7:** Correlation analysis: audit managers.

	**(1)**	**(2)**	**(3)**	**(4)**	**(5)**	**(6)**	**(7)**	**(8)**	**(9)**	**(10)**	**(11)**	**(12)**	**(13)**	**(14)**	**(15)**	**(16)**	**(17)**
IDEN_AUDIT	1																
TRUST_AUDIT	0.13^*^	1															
PSR_AUDIT	−0.17^*^	−0.07^*^	1														
GROWTH	−0.04	0.02	0.01	1													
INVENTORY	−0.01	0.04	−0.01	−0.03	1												
SIZE	0.07^*^	0.01	−0.03	0.00	0.12^*^	1											
LOSS	0.00	−0.07^*^	−0.04	−0.13^*^	0.01	−0.02	1										
AUDITOR_RESIGN	−0.01	0.03	−0.02	0.15^*^	−0.01	0.06^*^	0.02	1									
AUDITOR	0.03	0.03	−0.05	−0.03	−0.01	0.39^*^	−0.02	0.07^*^	1								
SOE	0.02	−0.03	0.02	−0.11^*^	0.11^*^	0.39^*^	0.13^*^	0.09^*^	0.15^*^	1							
DUAL	−0.02	−0.03	−0.01	0.01	−0.07^*^	−0.16^*^	−0.04	−0.03	−0.07^*^	−0.28^*^	1						
FIRST	0.01	0.05	−0.06	−0.03	0.05	0.22^*^	−0.03	0.07^*^	0.14^*^	0.19^*^	−0.04	1					
MAO	−0.03	0.02	0.04	−0.06^*^	−0.01	−0.01	0.19^*^	0.03	−0.01	0.05^*^	−0.00	−0.02	1				
INDEPEN	0.05	0.03	−0.05	−0.00	0.01	−0.05	0.01	0.01	0.03	−0.09^*^	0.12^*^	0.07^*^	0.03	1			
MANSHARE	−0.02	0.00	−0.05	0.08^*^	−0.11^*^	−0.36^*^	−0.12^*^	−0.03	−0.12^*^	−0.48^*^	0.23^*^	−0.10^*^	−0.06^*^	0.10^*^	1		
TRADE	0.01	0.06	0.03	0.05	−0.09^*^	−0.33^*^	−0.08^*^	0.02	−0.08^*^	−0.20^*^	0.11^*^	−0.04	−0.05	0.03	0.34^*^	1	
ROA	−0.00	0.09^*^	0.05	0.26^*^	−0.10^*^	0.02	−0.52^*^	0.04	0.04	−0.16^*^	0.05^*^	0.03	−0.30^*^	−0.03	0.17^*^	−0.14^*^	1

*Lower-triangular cells report Pearson's correlation coefficients, upper-triangular cells are Spearman's rank correlation*.

* *p < 0.05*.

*IDEN, Organizational identification; PSR, Parasocial relationship; TRUST, Integrity perception*.

*The larger the PSR, the weaker the PSR between the department heads and the CEOs*.

### Hypothesis Testing

The variance inflation factor (VIF) is far below 10, indicating that the multicollinearity concern is non-negligible. Thus, we conduct a regression analysis. Firstly, in the OLS regression, we test the main effect of PSR on OI. Secondly, due to the inaccuracy of a three-step method for testing the mediation effect and the non-robustness of the traditional Sobel test, we apply the bootstrapping mediation analysis method, which is considered relatively robust and accurate. We use the bootstrapping method for a mediation effect test by integrating the study of Wetzel et al. (2014). According to the mediation effect testing procedure by Zhao et al. (2010) and the mediator hypothesis testing method by Hayes (2013), we conduct the bootstrapping mediator test with 2,000 repetitions and a 95% confidence level.

Tables 8, 9 show the regression results for the main effect of a PSR. Without a mediator, the estimated effect of PSRs on organizational trust (OT) is −0.131 to −0.073 (*p* < 0.1 or 0.01). A large value of the PSR index indicates a weak PSR. Therefore, the main effect is significant; namely, the PSR between department managers and CEOs is positively associated with OI. H1 and H2 are supported.

**Table 8:** PSR and organizational trust.

**Variables**	**(1)**	**(2)**	**(3)**
	**TRUST_IC**	**TRUST_FINANCE**	**TRUST_AUDIT**
PSR_IC	−0.115^***^		
	(−3.37)		
PSR_FINANCE		−0.101^***^ (−4.75)	
PSR_AUDIT			−0.051^**^ (−2.25)
GROWTH	0.045 (1.38)	0.042 (1.43)	0.015 (0.36)
INVENTORY	0.009 (0.04)	0.008 (0.05)	0.450^***^ (2.66)
SIZE	0.014 (0.64)	−0.004 (−0.26)	0.010 (0.54)
LOSS	0.057 (0.57)	−0.055 (−0.84)	−0.098 (−1.29)
AUDITOR_RESIGN	−0.059 (−0.59)	−0.028 (−0.41)	0.094 (1.16)
AUDITOR	−0.013 (−0.13)	0.108 (1.20)	0.059 (0.62)
SOE	−0.050 (−0.85)	−0.018 (−0.43)	−0.090^*^ (−1.89)
DUAL	0.030 (0.42)	−0.013 (−0.35)	0.000 (0.01)
FIRST	0.000 (0.09)	0.001 (0.57)	0.001 (0.63)
MAO	0.056 (0.36)	0.102 (0.94)	0.284^**^ (2.28)
INDEPEN	0.560 (1.20)	−0.160 (−0.55)	0.050 (0.16)
MANSHARE	0.149 (0.52)	−0.028 (−0.31)	−0.073 (−0.79)
TRADE	−0.002 (−0.28)	−0.000 (−0.26)	0.004^**^ (2.23)
ROA	1.081^*^ (1.79)	0.397 (1.36)	0.555^*^ (1.79)
INDUSTRY	−0.010 (−1.22)	0.007 (1.35)	−0.004 (−0.60)
_cons	1.995^***^ (3.71)	2.663^***^ (6.98)	2.174^***^ (5.27)
*N*	424	1,105	902
R-Square	0.066	0.037	0.040
Adj.R-Square	0.03	0.02	0.02

*, **, and *** *are indicated to be significant at 10, 5, and 1%, respectively*.

**Table 9:** PSR and OI.

**Variables**	**(1)**	**(2)**	**(3)**	**(4)**	**(5)**	**(6)**
	**IDEN_IC**	**IDEN_** **FINANCE**	**IDEN_** **AUDIT**	**IDEN_IC**	**IDEN_** **FINANCE**	**IDEN_** **AUDIT**
PSR_IC	−0.073^*^ (−1.93)			−0.058 (−1.54)		
TRUST_IC				0.124^**^ (2.28)		
PSR_FINANCE		−0.069^***^ (−3.06)			−0.060^***^ (−2.65)	
TRUST_FINANCE					0.088^***^ (2.75)	
PSR_AUDIT			−0.131^***^ (−4.94)			−0.124^***^ (−4.70)
TRUST_AUDIT						0.131^***^ (3.31)
GROWTH	0.014 (0.39)	−0.011 (−0.35)	−0.123^**^ (−2.56)	0.009 (0.24)	−0.015 (−0.47)	−0.125^***^ (−2.62)
INVENTORY	−0.210 (−0.91)	−0.076 (−0.47)	0.018 (0.09)	−0.211 (−0.92)	−0.077 (−0.48)	−0.040 (−0.20)
SIZE	−0.005 (−0.20)	0.001 (0.04)	0.058^***^ (2.72)	−0.007 (−0.28)	0.001 (0.06)	0.057^***^ (2.67)
LOSS	0.006 (0.05)	0.006 (0.09)	0.179^**^ (2.01)	−0.002 (−0.01)	0.011 (0.16)	0.192^**^ (2.16)
AUDITOR_R~N	0.048 (0.43)	0.063 (0.87)	0.069 (0.72)	0.055 (0.50)	0.066 (0.90)	0.056 (0.60)
AUDITOR	−0.026 (−0.23)	−0.005 (−0.05)	−0.059 (−0.52)	−0.024 (−0.22)	−0.015 (−0.15)	−0.067 (−0.60)
SOE	−0.022 (−0.34)	−0.004 (−0.08)	−0.053 (−0.95)	−0.015 (−0.24)	−0.002 (−0.04)	−0.041 (−0.74)
DUAL	0.008 (0.10)	0.082^**^ (2.11)	−0.022 (−0.48)	0.004 (0.05)	0.083^**^ (2.15)	−0.022 (−0.49)
FIRST	−0.000 (−0.08)	0.000 (0.32)	−0.001 (−0.64)	−0.000 (−0.09)	0.000 (0.27)	−0.001 (−0.71)
MAO	0.126 (0.73)	0.088 (0.76)	−0.114 (−0.78)	0.119 (0.69)	0.079 (0.68)	−0.151 (−1.03)
INDEPEN	0.110 (0.21)	0.184 (0.59)	0.676^*^ (1.84)	0.040 (0.08)	0.198 (0.64)	0.669^*^ (1.83)
MANSHARE	0.223 (0.70)	0.026 (0.27)	−0.079 (−0.73)	0.204 (0.65)	0.029 (0.30)	−0.069 (−0.64)
TRADE	0.003 (0.46)	−0.000 (−0.16)	0.002 (1.08)	0.003 (0.49)	−0.000 (−0.13)	0.002 (0.83)
ROA	0.024 (0.04)	0.013 (0.04)	0.470 (1.29)	−0.111 (−0.17)	−0.022 (−0.07)	0.397 (1.09)
INDUSTRY	−0.003 (−0.30)	−0.005 (−0.81)	0.003 (0.36)	−0.002 (−0.17)	−0.005 (−0.92)	0.003 (0.43)
_cons	4.362^***^ (7.33)	4.246^***^ (10.48)	2.889^***^ (5.95)	4.114^***^ (6.83)	4.010^***^ (9.72)	2.605^***^ (5.31)
*N*	424	1,105	902	424	1,105	902
R-Square	0.019	0.017	0.056	0.031	0.024	0.067
Adj.R-Square	−0.02	0.00	0.04	−0.01	0.01	0.05

*, **, and *** *are indicated to be significant at 10, 5, and 1%, respectively*.

Table 10 displays the bootstrapping method results. For internal control managers, the percentiles for bootstrap and bias-corrected bootstrap analysis indicate that the direct effect is not significant because the CI of a direct effect is >0 (e.g., BC: [−0.13535, 0.0147845]). An indirect effect is significant because the CI of the indirect effect does not reach 0 (e.g., BC: [−0.0357188, −0.0022935]). This indicates that the organizational trust mediates the correlation between the PSR and the OI completely, given the insignificance of a direct effect. Similarly, for financial and audit managers, the CIs of direct and indirect effects do not reach 0. For example, the CI of direct and indirect effects in the financial department is [BC: −0.1102263, −0.0134493] and [BC: −0.0192012, −0.002345]. This indicates that the mediation path of “PSR–organizational trust–organizational identification” is significant and the mediation effect of organizational trust is partial. H3 is supported.

**Table 10:** Bootstrapping test for mediation effect.

**Bootstrap results**	**Observed Coef**.	**Bias**	**Bootstrap Std. Err**.	**[95% Conf. Interval]**	
**IC: Number of obs = 424, Replications = 1,999**					
Dir_eff	−0.058	−0.00085	0.038	−0.136	0.013 (P)
				−0.135	0.014 (BC)
Ind_eff	−0.014	0.00009	0.008	−0.033	−0.001 (P)
				−0.035	−0.002 (BC)
**FINANCE: Number of obs = 1,105, Replications = 2,000**					
Dir_eff	−0.060	0.00057	0.0255	−0.108	−0.012 (P)
				−0.110	−0.013 (BC)
Ind_eff	−0.008	0.00001	0.004	−0.017	−0.002 (P)
				−0.019	−0.002 (BC)
**AUDIT: Number of obs = 902, Replications = 2,000**					
Dir_eff	−0.124	0.00053	0.040	−0.206	−0.048 (P)
				−0.211	−0.054 (BC)
Ind_eff	−0.007	0.00011	0.004	−0.015	−0.00037 (P)
				−0.017	−0.001 (BC)

*P, percentile CI; BC, bias-corrected CI*.

### Robustness Test

To test the robustness of the above regression results, we use the two means to test the hypotheses. Firstly, the secretary of the board is one of the top executives in China and is usually the person in charge of the information disclosure of a company. We choose the variables on the PSRs between middle managers and secretaries of the board (*IC_PSR_2, FINANCE_PSR_2*, and *AUDIT_PSR_*2), which are derived using the same calculation method as substitution variables for PSRs between middle managers and CEOs. As shown in Tables 11, 12, the results are consistent with those previously reported in Tables 8, 9. Organizational trust completely mediates the correlation between PSRs and OI in the internal control department, but only partially mediates the relationship in financial and audit departments. Only the total effect in the internal control department is not significant (the estimated coefficient is −0.045 and the value of *p* is 0.2). One explanation is that the sample size for the internal control department is much less than that of financial and audit departments.

**Table 11:** Endogenous test: total effect.

**Variables**	**(1)**	**(2)**	**(3)**
	**IDEN_IC**	**IDEN_FINANCE**	**IDEN_AUDIT**
IC_PSR_2	−0.045 (−1.27)		
FINANCE_PSR_2		−0.069^***^ (−3.29)	
AUDIT_PSR_2			−0.140^***^ (−5.49)
GROWTH	0.023 (0.68)	−0.002 (−0.08)	−0.118^***^ (−2.64)
INVENTORY	−0.183 (−0.82)	−0.035 (−0.22)	−0.054 (−0.28)
SIZE	0.012 (0.51)	0.008 (0.47)	0.062^***^ (2.96)
LOSS	−0.061 (−0.59)	−0.028 (−0.41)	0.110 (1.27)
AUDITOR_RESIGN	0.058 (0.58)	0.076 (1.06)	0.089 (0.97)
AUDITOR	−0.068 (−0.65)	−0.020 (−0.21)	−0.037 (−0.34)
SOE	−0.004 (−0.06)	−0.036 (−0.82)	−0.027 (−0.48)
DUAL	0.016 (0.21)	0.070^*^ (1.83)	−0.036 (−0.81)
FIRST	−0.000 (−0.25)	0.000 (0.18)	−0.001 (−0.47)
MAO	0.129 (0.74)	0.115 (1.03)	−0.171 (−1.29)
INDEPEN	0.198 (0.40)	0.149 (0.48)	0.533 (1.47)
MANSHARE	0.217 (0.67)	0.006 (0.06)	−0.052 (−0.47)
TRADE	0.002 (0.39)	−0.000 (−0.14)	0.002 (0.84)
ROA_3	−0.172 (−0.27)	0.015 (0.05)	0.385 (1.10)
INDUSTRY	−0.005 (−0.51)	−0.003 (−0.54)	−0.001 (−0.14)
_cons	3.913^***^ (6.77)	4.106^***^ (10.38)	2.897^***^ (6.10)
*N*	442	1,132	914
R-Square	0.016	0.018	0.057
Adj.R-Square	−0.02	0.00	0.04

* and *** *are indicated to be significant at 10 and 1%, respectively*.

**Table 12:** Robustness test: mediation effect.

**Bootstrap results**	**Observed Coef**.	**Bias**	**Bootstrap Std. Err**.	**[95% Conf. Interval]**	
**IC: Number of obs = 442, Replications = 2,000**					
Dir_eff	0.030	−0.00103	0.038	−0.104	0.041 (P)
				−0.103	0.042 (BC)
Ind_eff	−0.014	0.00015	0.007	−0.031	−0.003 (P)
				−0.033	−0.004 (BC)
**FINANCE: Number of obs = 1,132, Replications = 2,000**					
Dir_eff	−0.061	0.00018	0.025	−0.113	−0.014 (P)
				−0.115	−0.016 (BC)
Ind_eff	−0.008	−0.00005	0.004	−0.016	−0.002 (P)
				−0.017	−0.002 (BC)
**AUDIT: Number of obs = 902, Replications = 2,000**					
Dir_eff	−0.128	0.00152	0.041	−0.207	−0.047 (P)
				−0.211	−0.054 (BC)
Ind_eff	−0.012	−0.00006	0.005	−0.023	−0.004 (P)
				−0.024	−0.005 (BC)

*P, percentile CI; BC, bias-corrected CI*.

Secondly, this study studies the influence of the PSR between middle managers and CEOs on their OI and the mechanisms of that process. To test the robustness of the regression results as mentioned earlier, we use the mean value (*TMT_IDEN*) of the organization identification of the CEO and the secretary of the board as a substitution variable for the dependent variable. The regression results of substitution variables are consistent with the previous ones. The regression results are displayed in Table 13.

**Table 13:** Robustness test.

**Variables**	**(1)**	**(2)**	**(3)**
	**TMT_IDEN**	**TMT_IDEN**	**TMT_IDEN**
PSR_IC	−0.162^***^ (−4.16)		
PSR_FINANCE		−0.186^***^ (−8.09)	
PSR_AUDIT			−0.069^***^ (−2.78)
GROWTH	−0.060 (−1.61)	−0.041 (−1.32)	0.006 (0.12)
INVENTORY	−0.036 (−0.15)	−0.156 (−0.96)	−0.036 (−0.19)
SIZE	0.030 (1.14)	0.022 (1.25)	0.015 (0.74)
LOSS	−0.164 (−1.42)	−0.032 (−0.45)	−0.032 (−0.38)
AUDITOR_RESIGN	0.142 (1.25)	0.046 (0.63)	0.058 (0.65)
AUDITOR	0.058 (0.50)	−0.001 (−0.01)	−0.158 (−1.50)
SOE	−0.061 (−0.91)	−0.069 (−1.53)	0.022 (0.42)
DUAL	0.123 (1.51)	0.043 (1.11)	0.030 (0.70)
FIRST	−0.003 (−1.54)	−0.001 (−0.90)	−0.001 (−0.40)
MAO	0.128 (0.72)	0.044 (0.38)	0.012 (0.09)
INDEPEN	0.595 (1.11)	0.683^**^ (2.18)	0.457 (1.33)
MANSHARE	−0.233 (−0.72)	−0.032 (−0.32)	0.002 (0.02)
TRADE	0.002 (0.31)	−0.001 (−0.35)	−0.000 (−0.10)
ROA	−0.183 (−0.26)	−0.043 (−0.14)	−0.053 (−0.16)
INDUSTRY	−0.011 (−1.12)	0.010^*^ (1.68)	0.009 (1.38)
_cons	3.713^***^ (6.00)	3.728^***^ (9.10)	3.816^***^ (8.37)
*N*	418	1,087	889
R-Square	0.080	0.075	0.018
Adj.R-Square	0.04	0.06	−0.00

*, **, and *** *are indicated to be significant at 10, 5, and 1%, respectively*.

## Conclusion and Implication

This study uses the survey data from the internal control research group of the CSRC in 2014 and tests the effect of a PSR on the OI of middle managers and its mediation path. The results imply that the PSR between middle managers and CEOs positively predicts the OI of middle managers, and the organizational trust of middle managers mediates that relationship. In particular, organizational trust completely mediates the correlation between the PSR and OI of internal control managers but partially mediates that relationship for financial and audit managers.

This study has the following implications regarding the effect of the PSR between middle managers and CEOs on the OI of middle managers.

Firstly, PSRs can be leveraged as a vehicle for building OT and OI in the workplace, and CEOs should be actively working to strengthen these relationships. Previous studies have shown that OI can have many positive effects, such as improving job satisfaction, increasing organizational citizenship behavior (Tse et al., 2014), and increasing audit independence and quality (Bauer, 2015). Organizational members with high OI are more committed to their work as well as exhibit positive attitude and behavior (Karanika-Murray et al., 2015), such as improving their superior–subordinate relationship, increasing their organizational citizenship behavior (Zhang and Chen, 2013), increasing their job satisfaction (Karanika-Murray et al., 2015), enhancing their firm output (Lange et al., 2015), and reducing the costs of their agency (Boivie et al., 2011; Lange et al., 2015). Because the PSR between middle managers and CEOs originates from the emotional resonance induced by the language and behavior of CEOs, CEOs can utilize various channels, such as video conference, Facebook, Twitter, Youtube, and public speech, to convey positive personal characteristics, views, attitudes, and caring signals; attract the attention and preferential affection of middle managers and other employees; and then build and maintain a PSR, which may lead to more positive cognition and behaviors. In addition, future research could investigate specific pathways by which CEOs or other C-suite can strengthen PSR in their workplace.

Secondly, CEOs should value the influence of PSR and organizational trust and improve his/her own social and work abilities. PSR may be used as a tool for building better organizations, increasing differentiation, sustaining superior competitive advantage, enhancing talent development, etc. Organizational trust of middle managers may have a significant positive impact on their sense of responsibility, ethical behavior, organizational commitment, job satisfaction, and performance (Williams and Anderson, 1991; Agnihotri and Krush, 2015). Our study reveals that the trust relationship between CEOs and middle managers is significantly positively correlated with the OI of the middle managers. It is critical for the CEOs to establish a good and credible image because the trust of middle managers in CEOs is based on the perceptions and assessments of middle managers on the ability of the CEOs to do things and to implement goodwill (Williams and Anderson, 1991; Mayer et al., 1995; McAllister, 1995; Agnihotri and Krush, 2015). CEOs can respond by improving their appearance, speech, ability, act, and the ways they treat their middle managers to improve PSRs or perceptions of trust of middle managers.

Finally, CEOs should adopt different methods to influence different managers. Organizational trust completely mediates the relationship between the PSR and OI but only partially mediates the relationship in financial and audit departments. For internal control managers, CEOs should take measures that only affect the PSR and organizational trust of the middle managers, but there may need more comprehensive measures for financial and audit managers. In this study, we use surveys over 2,500 listed firms and 12,000 individuals with a response rate of ~85%. Therefore, our results could generalize to most of the publicly listed firms in the emerging markets of China. However, our study is not free of limitations. Firstly, there is a significant SD when measuring the PSR between MMs and CEOs, which indicates the nature to which PSRs are experienced is highly variable and dependent on both personal and contextual factors. This big SD likely limits the strength to which conclusions as to the affect can be made. Secondly, the sampled population consists of a disproportionately large cohort of individuals from finance and audit departments. Given the influence of in-role contextual factors on the nature to which PSR is experienced, this likely skews the findings to be more reflective of PSR affection within this department.^1^ Future research could further explore the economic consequences of PSRs and/or how PSRs could be used to enhance workplace practice. It would be beneficial to investigate current workplace trends and how PSR can be used as a tool for building better organizations, increasing differentiation, sustaining superior competitive advantage, and enhancing talent development. In addition, more research work is expected in the future to ground these findings in a larger organizational context. For instance, an interesting question would be whether it is expected that the nature of PSR and its effects on OT and OI would be the same in a different geographic context or whether it would vary over time.

## Data Availability Statement

The original contributions presented in the study are included in the article/supplementary material, further inquiries can be directed to the corresponding author/s.

## Author Contributions

YL: idea, writing manuscript, and running data. BL: funding, revising manuscript, and advice. HZ and XY: revising manuscript and advice. All authors contributed to the article and approved the submitted version.

## Funding

This work was supported by Philosophy and Social Science Foundation of China: 18VSJ082 and National Natural Science Foundation of China: Nos. 71332004, 71272198, and 71862017.

## Conflict of Interest

The authors declare that the research was conducted in the absence of any commercial or financial relationships that could be construed as a potential conflict of interest.

## Publisher's Note

All claims expressed in this article are solely those of the authors and do not necessarily represent those of their affiliated organizations, or those of the publisher, the editors and the reviewers. Any product that may be evaluated in this article, or claim that may be made by its manufacturer, is not guaranteed or endorsed by the publisher.

## Footnotes

^1^We owe our thanks to the anonymous reviewers for making these insightful comments on the strength and weakness of our study.
